# Nanoparticle Based Cardiac Specific Drug Delivery

**DOI:** 10.3390/biology12010082

**Published:** 2023-01-04

**Authors:** Dong Li, Yura Son, Michelle Jang, Shu Wang, Wuqiang Zhu

**Affiliations:** 1 Department of Cardiovascular Diseases, Physiology and Biomedical Engineering, Center for Regenerative Medicine, Mayo Clinic Arizona, Scottsdale, AZ 85259, USA; 2 Department of Cardiology, Dongfang Hospital, The Second Affiliated Hospital of Beijing University of Chinese Medicine, Beijing 100078, China; 3 Ira A. Fulton Schools of Engineering, Arizona State University, Tempe, AZ 85281, USA; 4 College of Health Solutions, Arizona State University, Phoenix, AZ 85004, USA

**Keywords:** cardiac, targeted delivery, myocardial, ischemia, nanomaterial, nanoparticle

## Abstract

**Simple Summary:**

Heart disease is the leading cause of death, globally. Cardiomyocytes in adult mammalian hearts lose their capacity to divide. Therefore, the loss of heart muscles after a heart attack is usually irreversible. Many novel therapies that aim to repair injured myocardium have been reported in the last few decades. In this field, there is an urgent need to develop a targeted drug delivery system that specifically delivers therapeutic agents to injured hearts. Nanoparticles are the most commonly used vehicles for targeted drug delivery in preclinical and clinical studies. Here, we provide the field with a comprehensive review of the latest strategies involving nanoparticle-based cardiac-specific drug delivery for the treatment of heart diseases.

**Abstract:**

Heart failure secondary to myocardial injuries is a leading cause of death worldwide. Recently, a growing number of novel therapies have emerged for injured myocardium repairment. However, delivering therapeutic agents specifically to the injured heart remains a significant challenge. Nanoparticles are the most commonly used vehicles for targeted drug delivery. Various nanoparticles have been synthesized to deliver drugs and other therapeutic molecules to the injured heart via passive or active targeting approaches, and their targeting specificity and therapeutic efficacies have been investigated. Here, we summarized nanoparticle-based, cardiac-specific drug delivery systems, their potency for treating heart diseases, and the mechanisms underlying these cardiac-targeting strategies. We also discussed the clinical studies that have employed nanoparticle-based cardiac-specific drug delivery.

## 1. Introduction

Heart failure after myocardial injuries is a leading cause of death, worldwide. Cardiomyocytes in adult mammalian hearts lose the capacity to proliferate. Hence, the loss of myocardium due to injuries such as acute myocardial infarction (MI) often results in fibrotic scarring and depressed cardiac functions. Recently, there has been a rapid growth of novel therapies that have aimed to repair injured myocardium, such as cell-based therapy [1], gene engineering [2], and novel medications with anti-inflammatory [3] and proangiogenic [4] effects. Despite these encouraging discoveries, the systemic administration of these therapeutic agents often leads to nonspecific effects due to whole-body distribution. The targeted delivery of these therapeutic agents to the injured heart is an area of active research in the cardiovascular field [5,6]. At present, the approaches for cardiac-specific drug delivery include intracoronary delivery [7,8], pericardial delivery [9], epicardial delivery [10,11], intramyocardial delivery [12], and implantable device-based delivery [13,14]. Although these approaches increase the local distribution and concentration of therapeutic agents in the heart, their application often requires invasive procedures.

The use of targeted drug delivery systems is always necessary as they provide unique advantages to enhance efficacy and reduce off-target effects. Nanoparticles (NPs) are the most common vehicles to deliver therapeutic materials to the target tissue. For the delivery of therapeutic agents, NPs can be composed of a variety of nanomaterials and structures, including lipids, polymers, dendrimers, carbon nanotubes, and metallic nanoparticles [15,16]. NPs have gained much attention in the cardiovascular field owing to their unique biological properties and biocompatibilities. In comparison to other vectors, nanomaterials possess unique properties, including controlled and sustained release, reduced drug degradation, side effects, and increased in vivo efficacy [17]. In addition, the small sizes (1–100 nm) of NPs can cross cell membranes and deliver therapeutic materials to intracellular compartments [15]. Furthermore, the surface of NPs can be modified to increase their affinity to specific cell surfaces [18,19]. The development of targeted NP delivery systems also enables multiple treatments, such as small molecules, nucleic acids, and peptides, etc., to be delivered together to specific organs or cells [20]. Recent studies have shown that NPs formulated with therapeutic agents attenuate post-MI myocardium damage [21,22,23,24]. In this paper, we discuss the most recent publications concerning NP-based, cardiac-specific drug delivery systems and summarize their applications in preclinical and clinical studies for the treatment of ischemic heart disease. We searched PubMed with the following keywords: “cardiac” AND “targeted delivery” AND “myocardial” AND “ischemia” AND “nanomaterial” OR “nanoparticles”. The publication dates range between 2010 and 2022.

## 2. Types of Nanocarriers

**Figure 1:**
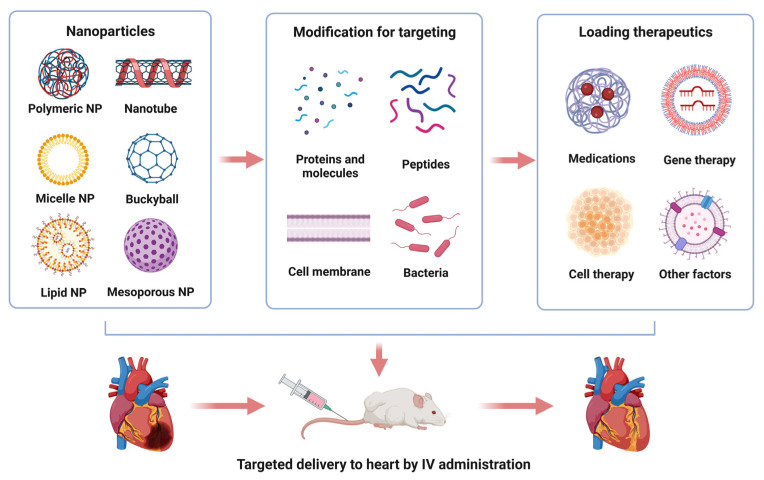
Illustration of nanoparticle-based drug delivery for the treatment of myocardial injuries. Approaches to process nanoparticles for cardiac-specific delivery of therapeutic agents (**upper** panel). Intravenous administration of cardiac-targeting nanoparticles for myocardial regeneration and repair (**lower** panel). Note: we used nanosphere in this illustration to represent polymeric NPs.

NPs are typically defined as particles with diameters of less than 100 nanometers (nm) that are used for diagnostic or therapeutic purposes [25]. Particles smaller than 10 nm or larger than 200 nm will be quickly filtered by the kidney and accumulated in the liver and spleen after entering the blood circulation [26]. Properties of NPs, such as size, lipophilicity, and surface charge, are the determining factors for targeted delivery [27]. Along with the advances in bioengineering, significant novel materials and methods have emerged for synthesizing NPs. Sorted by materials, NPs can be classified as either organic, inorganic, or hybrid particles [15]. Organic NPs include proteins [28], carbohydrates [29], lipids [30], polymers [31], and other organic compounds [32] (Figure 1). Among the organic NPs, liposome is the first FDA-approved particle for drug delivery owing to its high biocompatibility [33,34]. Poly lactic-co-glycolic acid (PLGA) is another FDA-approved material for drug delivery [35]. Inorganic NPs include the carbon-based particles, such as carbon nanotubes [36], buckyballs [37], graphene [38], and metal particles, which are made of gold [39], silver [40], or iron oxide [41]. Because of their physical, electrical, magnetic, and optical properties, inorganic NPs possess unique advantages in tailoring them to the specific functions of the targeted cells or organs [42]. Moreover, a myriad of hybrid NPs, which combine the advantages of organic and inorganic NPs, can extend the release time and promote the specificity and efficiency of drugs [43]. For example, the fusion of liposomes and magnetic NPs forms magnetoliposomes (MLs) [44], deoxyribozyme (DNAzyme) functionalized NPs [45], and molecularly organic-inorganic hybrid hollow mesoporous organosilica NPs (HMONs) [46]. As the field of cardiac-specific targeting NPs evolves rapidly, all three types of particles have been utilized to develop the cardiac-targeted drug delivery system [26].

## 3. Targeting Strategies

NP-based targeting strategies can be roughly categorized into two classes, namely, passive targeting and active targeting. Passive targeting is based on the physicochemical properties of the cells and microenvironment under certain circumstances. For example, the release of cytokines from the damaged tissues increases the local vascular permeability and inflammation responses, resulting in the enhanced permeability and retention (EPR) effect [47]. Recent studies have demonstrated that NPs can be delivered to the myocardium by the EPR effect in the post-MI heart [48,49,50]. However, the vessel permeability is normalized within a few days [51], which indicates that the EPR effect does not provide a long-term option for the passive targeting of NPs to injured myocardium. Alternatively, the passive targeting of NPs can be accomplished by using NP’s capability of covalently coupling to circulatory cells after surface modification [52]. The surface modification enables NPs to bind to specific cells in the peripheral blood and travel to the hearts [53,54]. In contrast to passive targeting, the active targeting strategy utilizes cell-specific ligands that enable specific and highly efficient binding to the surface receptors/transporters of cardiomyocytes or other cells in the heart [55,56]. For example, NPs can conjugate with the surface proteins of cardiomyocytes, specific antibodies, small peptides, or cytokines that serve as biomarkers for MI [57,58].

## 4. Cell Type-Specific Targeting

Various types of cells, such as cardiomyocytes, fibroblasts, and inflammatory cells, etc., are involved in pathological left ventricular remodeling in post-MI heart [59]. Accordingly, cardiac-specific drug delivery may be achieved by targeting these cells. In this section, we summarize the cardiac-targeted delivery studies, classifying them by different targeted cells and sites (Table 1).

### 4.1. Cardiomyocytes

Cardiomyocytes in adult mammalian hearts exit the cell cycle, suggesting that their capability to regenerate myocardium after injuries is minimal. Therefore, salvaging the ischemic myocardium is an important strategy for the treatment of MI [60,61]. There are several receptors in the cardiomyocyte membrane that were targeted to deliver therapeutic NPs to ischemic hearts. It is known that the expression of the angiotensin II type 1 receptor (AT_1_R) is increased in the membrane of cardiomyocytes in the ischemic myocardium. Therefore, AT_1_R has been utilized to develop cardiac-specific targeting therapy for the early stage of ischemia [62,63,64]. Xue et al. developed an NP with AT_1_ peptides on the NP surface, which binds to the AT_1_R in the cardiomyocyte membrane in the ischemic myocardium. The anti-miRNA-1 antisense oligonucleotide (AMO-1) was delivered to cardiomyocytes in the mice after myocardial infarction using the AT1 peptide-conjugated NPs (AT1-AMO-1-NPs) to inhibit apoptosis [65]. After intravenous injection, AT_1_-AMO-1-NPs were highly accumulated in the ischemic myocardium during the desired early period. In comparison to the group without AT_1_ peptide (AMO-1-NPs), the treatment of AT_1_-AMO-1-NPs showed a cardioprotection capacity by inhibiting apoptosis in the infarct border zone of the post-MI mice and reducing the infarct area. Despite the increased expression in the ischemic myocardium, AT_1_R is also expressed in other organs, such as the liver, lung, adrenal gland, and kidney [66]. Therefore, more studies are warranted to prove the specificity of AT_1_R-based cardiac-specific drug delivery.

To obtain more specific cardiac-targeting ligands, a phage display technique has been applied to isolate peptides with a high affinity to cardiomyocytes. With this technique, McGuire et al. identified a peptide in the PCM.1 phage (WLSEAGPVVTVRALRGTGSW) that bound to isolated primary cardiomyocytes more than the control peptide [67]. This phage peptide contains a 12 amino acid segment with a sequence identical to a peptide in tenascin-X (TNX). TNX is a member of the tenascin family and is expressed in the connective tissue of the heart [68]. The real-time PCR assay showed that the localization of the PCM.1 phage was significantly improved in cardiomyocytes compared to other tissues. In addition, the PCM.1 phage displayed an increased binding affinity to the heart tissue in vivo compared to the phages containing other peptides. These data suggest that targeting the TNX receptor might be a potential approach for delivering drugs specifically to the cardiomyocytes.

Cell membrane-coating technology has been applied to the synthesis of NPs. The technology allows NPs to possess the characteristics of natural cells [69,70]. Previous research has confirmed that macrophages can bind to the vascular cell adhesion molecule-1 (VCAM-1), which is highly expressed in cardiomyocytes during ischemia and reperfusion [71]. To mimic the interaction between cardiomyocytes and macrophages, Wei et al. established a biomimetic nanoplatform consisting of a PDA core and a macrophage membrane shell (PDA@M-NPs) [72]. With the expression of α4β1 integrin on the surface of the macrophage membrane shell, the PDA@M-NPs inherit the intrinsic properties of the macrophage membrane. The PDA@M-NPs displayed significantly higher affinity to cardiomyocytes that are challenged by hypoxia and reperfusion compared to the NPs without a macrophage membrane-coating. In addition, the PDA@M-NPs showed cardioprotective effects, owing to their antioxidative and antipyroptosis capacity after intravenous injection to post-MI mice.

Exosomes are the natural nanoscale extracellular vesicles that carry multiple signaling biomolecules, including protein and nucleic acids [73]. As extracellular organelles that mediate the systemic information exchange and long-distance interactions between cells, exosomes and their applications in heart failure treatment have been extensively studied for decades [74,75]. Liu and colleagues developed magnetic NPs for targeting exosomes from the cardiomyocytes after MI. The NPs consisting of a Fe_3_O_4_ core and a silica shell were modified with two antibodies that bind to either the CD63 antigen on the surface of the exosomes or to the myosin light chain surface makers in the injured myocardium [76]. Upon the application of a local magnetic field, the accumulation of NPs and the cleavage of the hydrazone bonds under the acidic pH of injured cardiac tissue resulted in the local release of exosomes from the NPs. The magnetic-guided accumulation of the captured CD63-expressing exosomes in infarcted myocardium led to the improvement of heart function and the reduction in infarct size after intravenous injection in post-MI rabbits and rats.

The endocardium, the inner layer of the heart muscle, expresses atrial natriuretic peptide receptors. Santos and colleagues developed PEGylated porous silicon NPs that were conjugated to atrial natriuretic peptides [77]. The intravenous injection of the atrial natriuretic peptide-conjugated NPs in post-MI rats showed a higher accumulation in the endocardial layer of the left ventricle. This suggests that conjugating them to atrial natriuretic peptides may target the NPs to the endocardium of the heart. Yu and colleagues developed adenosine-loaded lipid NPs that were conjugated to atrial natriuretic peptide [78]. The atrial natriuretic peptide-conjugated NPs were significantly accumulated in the infarct endocardium of rats after their intravenous injection and reduced the infarct size compared to the unconjugated NPs.

### 4.2. Vascular Endothelium

Acute MI induces vascular endothelial damage and the exposure of the components of the subendothelial matrix, including collagen, fibronectin, and the von Willebrand factor (vWF), resulting in the recruitment of platelets to the injured area [79,80]. Thus, platelets may act as natural “ligands” that bind to injured endothelium after MI. Li et al. formulated an NP with poly(5,5-dimethyl-4,6-dithiopropylene glycol azelate) (PTK) and cyclosporine A, and coated the NP with platelet membrane [81]. Following their intravenous injection to post-MI mice, the NPs specifically bound to the injured endothelium of the infarcted myocardium; this resulted in the reduction of inflammation, reactive oxygen species, and myocardial fibrosis, and improved left ventricular remodeling [81]. Zhou et al. developed platelet membrane vesicles (PMVs) that encapsulated Carvedilol, a nonselective β-blocker, in the PMVs (PMVs@Carvedilol). The PMVs@Carvedilol was significantly accumulated in the damaged vascular endothelium of the ischemic myocardium in post-MI rats. In addition, the treatment with PMVs@Carvedilol showed a reduction in cardiomyocyte apoptosis and the infarct size, and preserved heart function [82]. Tang et al. fused the membrane of cardiosphere-derived cardiac stem cells (CSCs) with platelet nanovesicles that express platelet surface markers [83]. The platelet nanovesicle-fused CSCs selectively bound to the collagen-coated surfaces and endothelium-denuded aortas and reduced the infarct size in post-MI rats.

### 4.3. Monocytes

**Figure 2:**
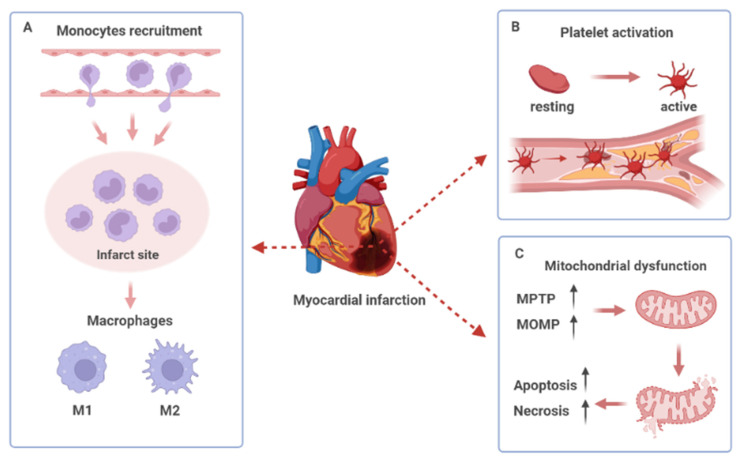
Pathological left ventricular remodeling post-myocardial infarction. (**A**) The recruitment of monocytes to infarct myocardium and the phenotype switching of macrophages. (**B**) Platelet activation, adhesion, and aggregation in the damaged site of the vascular endothelium. (**C**) Opening of the mitochondrial permeability transition pore (MPTP) and increased mitochondrial outer membrane permeability (MOMP) lead to mitochondrial dysfunction and cell death.

After MI, the pro-inflammatory cytokines released from the damaged cardiomyocytes attract the circulating immune cells, such as neutrophils and monocytes, to the injured heart [84]. In the injured myocardium, the phenotypic transition of macrophages from anti-inflammatory M2 to pro-inflammatory M1 accelerates the inflammatory response, leading to aggravated infarction, left ventricular remodeling, and cardiac dysfunction [85]. The recruitment of monocyte to the ischemic myocardium has been utilized as a passive targeting approach for drug delivery to injured heart tissue (Figure 2A). As an AT_1_R blocker, irbesartan has been widely used to treat hypertension and heart diseases. Nakano et al. developed bioabsorbable PLGA-NPs formulated with irbesartan (irbesartan-NPs) [86]. Due to the enhanced vascular permeability during myocardial ischemia and reperfusion, the irbesartan-NPs were rapidly taken up by the circulating monocytes via the EPR-effect and were enriched in the injured myocardium during monocyte recruitment. As a result, the intravenous administration of irbesartan-NPs reduced the infarct size and ameliorated ventricular remodeling in the mouse model of myocardial ischemia and reperfusion. Pioglitazone is a peroxisome proliferator-activated receptor-gamma (PPARγ) agonist with unique anti-inflammatory effects on monocytes and macrophages. The intravenous injection of PLGA NPs formulated with pioglitazone showed cardioprotective capacities in the mouse models of myocardial ischemia and reperfusion [87]. In addition, the pioglitazone-NPs were predominantly delivered to circulating monocytes and macrophages in the injured animal hearts, resulting in the inhibition of monocytes activation, macrophage-mediated acute inflammation, and the improvement of cardiac healing after MI [87].

In addition to the EPR effect of passive targeting, conjugating NPs with ligands that bind to the specific receptors of monocytes represents an active targeting approach for drug delivery to injured heart tissue [55,56]. This approach exploits the ability of monocytes to actively migrate to injured sites and release drugs upon reaching these sites. For example, the Ly-6C^high^ inflammatory monocytes originated from the spleen were recruited to the post-MI heart and promoted inflammation through a C-C chemokine receptor type 2 (CCR2)-dependent manner in mice [88]. Wang et al. developed the PEG-distearoylphosphatidylethanolamine lipid micelles formulated with a CCR2 antagonist and conjugated them with an anti-CCR2 antibody [89]. These micelles exhibited a high affinity to CCR2-positive macrophages and reduced the number of inflammatory monocytes in both the heart and the spleen of the mice. In addition, the micelles inhibited the inflammatory monocyte infiltration into the infarcted myocardium, significantly reduced the infarct size and improved cardiac function in the post-MI mice [89]. Hyaluronan (HA) is an anionic, nonsulfated glycosaminoglycan and an important component of the extracellular matrix (ECM). HA binds to the CD44 and CD168 receptors in the membrane of monocytes [90,91]. Ben-Mordechai et al. developed lipid-based NPs containing HA (HA-LPs) to target inflammatory macrophages in the infarct area [92]. The HA-LPs were significantly accumulated in the infarct heart more than in the other organs in the post-MI mice. Hemin is an iron-containing porphyrin that activates heme oxygenase-1, an enzyme with anti-inflammatory and cytoprotective properties. The authors encapsulated hemin to the macrophage-targeting HA-LP (hemin/HA-LP). Following its intravenous injection to the post-MI mice, the hemin/HA-LP effectively and specifically targeted the macrophages in the infarct heart and promoted the phenotypic switch of the macrophages from M1 (pro-inflammatory) to M2 (anti-inflammatory), which led to alleviated ventricular remodeling and increased the cardiac function in the mice [92]. Together, the data suggests that the modulation of the macrophage phenotype can be a promising strategy for promoting cardiac healing.

The development of Cell Systematic Evolution of Ligands by EXponential enrichment (Cell-SELEX) enables the screening of the ligands with a high affinity to specific cells [93]. With this technique, Huang and colleagues identified an aptamer (designated as J10 aptamer) that has a high binding affinity to monocytes [94]. The authors developed J10-conjugated lipid NPs and injected them into mice with ischemia and reperfusion. The J10-conjugated lipid NPs displayed a high binding affinity to monocytes and a minimal affinity to endothelial cells in the heart compared to NPs without modification. The authors further encapsulated IOX2 to J10-conjugated lipid NPs and demonstrated that the intravenous injection of the NPs improves the heart function in mice with ischemia and reperfusion. Taken together, cell membrane coating may be utilized to modify the surface of NPs and enhance their binding affinity to specific targeting sites [69,70].

### 4.4. Platelets

Platelet is known to interact with the monocytes during MI through the binding between the P-selectin and the P-selectin glycoprotein ligand-1(PSGL-1) [95]. As part of the family of cell adhesion molecules, PSGL-1 is highly expressed in circulating Ly6C^high^ monocytes and the ischemic myocardium [96,97,98]. Cheng et al. established a platelet-like proteoliposomes (PLPs) system to mimic the interactions between platelets and circulating monocytes [99]. To achieve a high binding affinity with monocytes, several platelet membrane proteins (PMPs) were used to fabricate PLPs, including CD62P, GPIIb, and CD42c, which are known to mediate the binding of platelets with monocytes [100]. The intravenous injection of PLPs showed a significantly higher accumulation at the infarct myocardium compared to the liposomes without PMP modification in the post-MI mice. Tan et al. developed a protocol to coat mesoporous silica nanospheres with platelet-like fusogenic liposomes [101]. The system delivered miR-21, an anti-inflammatory agent, to inflammatory monocytes in the blood circulation in mice with myocardial ischemia and reperfusion by binding it to the membrane proteins of the platelets, such as P-selectin, CD42c, CD47, integrin GP IIb/IIIa, and GP Ib𝛼 [101]. As a result, miRNA-21 entered the cytoplasm of the inflammatory monocytes through membrane fusion and reprogrammed the monocyte-derived inflamed macrophages, resulting in a significant reduction in the levels of inflammatory factors and the preservation of the cardiac function in post-MI mice.

The activation, adhesion, and aggregation of platelets all play a critical role in the development of atherosclerotic and acute thrombotic events, which are the risk factors of MI [102] (Figure 2B). Thrombolytic therapy is an important treatment in rebuilding blood flow in patients with MI, particularly for patients with ST-segment elevation [103]. However, the timely removal of blood clots is a big challenge considering the dense fibrin networks. Xie et al. assembled an *E. coli* Nissle 1917 (EcN) into nanotubes to deliver urokinase (uPA), the most commonly used thrombolytics [104]. Fucoidan is a type of highly sulfated polysaccharide containing l-fucose groups that exhibit a high affinity for P-selectin, mimicking its main ligand, the P-selectin Glycoprotein Ligand 1 (PSGL-1). To accurately deliver uPA to the thrombus, fucoidan was used to coat the surface of nanotubes. Fucoidan selectively interacts with P-selectin on the surface of activated platelets in the thrombus and exhibits various biological benefits, such as anticoagulants and antithrombotics [105]. With the driven motion of EcN, the nanotubes significantly interact with the activated platelets, promoting uPA accumulation in the thrombus, thereby conferring a better thrombolytic effect. Thus, the synergy between bacteria-driven motion and the specific ligand may provide a promising thrombolytic treatment strategy for MI.

It was reported that miRNA-133 is a cardioprotective molecular in post-MI animals [106,107,108]. Sun et al. developed an NP with polyethylene glycol (PEG) and polylactic acid (PLA) to deliver miRNA-133 [109]. Arginine-glycine-aspartic acid tripeptide (RGD) was conjugated with the NP (RGD-PEG-PLA-NP) as a ligand, which conferred a high binding affinity to the activated platelets in the thrombus but not the circulating platelets [110]. The RGD-PEG-PLA-NP delivered miRNA-133 to the infarct myocardium in the post-MI rats by the platelet-targeting effect of RGD through intravenous injection, leading to a reduction in cardiomyocyte apoptosis and the myocardial infarct size, as well as preserved cardiac function [109].

### 4.5. Mitochondria

In addition to ATP production, mitochondria play significant roles in the metabolism and homeostasis of the cardiomyocytes. During MI, oxidative stress and the change in pH triggers the opening of the mitochondrial permeability transition pore (MPTP), which results in mitochondrial dysfunction and the activation of the mitochondrial apoptosis pathway [111,112] (Figure 2C).

Cyclosporin A (CsA) is a well-known inhibitor of MPTP. Ikeda et al. generated PLGA-NPs loaded with CsA (CsA-PLGA-NPs) [113]. Due to the increased permeability of the cells at the stage of reperfusion, the concentration of CsA-PLGA-NPs increased in the cardiomyocytes, particularly in the mitochondria after intravenous injection to mice with myocardial ischemia and reperfusion. Inhibiting the opening of the MPTP by CsA protected the heart from ischemia and reperfusion injuries by maintaining the structure and function of mitochondria and mitigating ventricular remodeling. Zhang et al. synthesized a type of PLGA-PEG-NPs loaded with CsA, as well (CsA-PLGA-PEG-NPs) [114]. To deliver CsA precisely to the mitochondria of the cardiomyocytes, the peptide Szeto-Schiller 31 (SS31) was used as a ligand to specifically bind to cardiolipin, an anionic phospholipid expressed on the inner mitochondrial membrane [115,116]. The intravenous injection of the mitochondria-targeted NPs significantly reduced cardiomyocyte apoptosis and the infarct size in the rat model of myocardial ischemia and reperfusion compared to the CsA-NPs without SS31 modification.

In addition to the opening of the MPTP, the mitochondria outer membrane permeabilization (MOMP) is another mechanism of mitochondrial-mediated cell death [117]. Ishikita and colleagues formulated PLGA-NPs with the mitochondrial division inhibitor 1 (Mdivi1-NPs) [118]. The Mdivi1-NPs were significantly accumulated in the cytosol and mitochondria of the H_2_O_2_-pretreated cardiomyocytes. Following their intravenous injection, the Mdivi1-NPs reduced the infarct size and decreased cardiomyocyte apoptosis in the mice with ischemic myocardial injury. These cardioprotective effects were attributed to not only the MPTP inhibition by Mdivi1 but also the MOMP inhibition via the downregulation of the Drp1/Bax pathway. Although this study demonstrated that NPs were abundantly taken up by the mitochondria of the cardiomyocytes under the hypoxia condition, in vivo biodistribution studies are necessary to confirm the specific targeting effect of Mdivi1-NPs to the heart versus other organs.

The delivery of active ingredients from natural plants with NPs provides a new strategy for the treatment of MI. Tanshinone IIA (TN) is a major active ingredient of Salvia miltiorrhiza, which is widely applied to the treatment of cardiovascular disease in Asia [119]. Zhang and colleagues developed lipid-polymeric NPs to deliver TN [120]. To increase their targeting specificity and therapeutic efficacy, the NPs were conjugated with triphenylphosphonium (TPP), which can readily pass the lipid bilayers and be accumulated within the mitochondria through its high negative membrane potential. In comparison to free TN and TN-NPs without ligand conjunction, the TN-TPP-NPs showed an increased uptake rate and bioavailability in the heart after their intravenous injection to post-MI rats. The treatment of TN-TPP-NPs significantly reduced the infarct size. Similarly, Wang J et al. developed Baicalin (BA)-laden lipid polymer NPs that were conjugated with atrial natriuretic peptide and TPP [121]. BA is a bioactive compound extracted from plants that has been shown to attenuate ischemic injury by inhibiting mitochondrial-mediated apoptosis via the reduced activation of the mitogen-activated protein kinase pathway [122]. The intravenous injection of the BA-TPP-NPs to post-MI rats exhibited a greater myocardial distribution, longer drug release time, and higher potency of reducing the infarct size compared to the control NPs [121].

Cyclic arginine-glycine-aspartic (RGD) peptides can specifically bind to αvβ3 integrin, which is highly expressed in the infarcted myocardium as part of the angiogenesis process [123,124]. Dong et al. exploited the lipid-polymer NPs conjugated with cyclic RGD peptide to deliver TN and calycosin (CAL) to the injured myocardium [125]. Following their intravenous injection to post-MI mice, the RGD-TN-CAL-NPs displayed a prolonged drug release time and increased drug accumulation in the mitochondria of the cardiomyocytes, resulting in a reduced infarct size [125].

Nanozymes are nanomaterials with intrinsic enzyme-like characteristics. Nanozymes are capable of scavenging cytotoxic free radicals by mimicking the activity of natural enzymes, such as peroxidase (POD) [126], SOD [127], and catalase (CAT) [128]. Zhang and colleagues developed an enzyme-mimicking nanozyme consisting of recombinant human ferritin nanocage (FTn) with an MnO_2_ fenozyme core [129]. After TPP modification, the TTP-FTn-nanozyme was equipped with mitochondria-targeting capability, which overcame the intracellular lysosome barrier. After its intravenous injection to the post-MI mice, TTP-FTn-nanozyme attenuated the oxidative damage in the heart with reduced the infarct size and preserved cardiac function. Due to its high stability and durability, various biological functions, and low cost, nanozyme has shown a broad applicable potential in the field of regenerative medicine [130].

## 5. ECM Proteins

Matrix metalloproteinases (MMPs) are a family of enzymes in the ECM of the myocardium, and MMP-2 and MMP-9 are up-regulated in the myocardium after MI [131]. Nguyen et al. generated spherical micellar NPs conjugated with enzyme-responsive peptide-polymer amphiphiles (PPA-NPs) [132]. The PPA-NPs underwent a morphological transition from spherical-shaped, discrete materials to network-like assemblies upon cleavage by MMP-2 and MMP-9. The PPAs-NPs, after their intravenous injection, were accumulated in the damaged myocardium of the post-MI mice, particularly in the infarct zone, but not in the remote zone with viable myocardium. Additionally, the enzyme-responsive NPs showed long-term retention (up to 28 days) in the infarct myocardium.

When tissue damage causes bleeding, fibrinogen is converted into fibrin at the wound area by thrombin, a clotting enzyme. It was reported that thymosin beta 4 (Tβ4) confers cardioprotection in injured hearts, while its efficiency is limited by the low local concentration within the infarct myocardium [133,134]. Huang et al. established a Tβ4 delivery system by conjugating Tβ4 and cysteine-arginine-glutamic acid-lysine-alanine (CREKA), a fibrin-targeting moiety clot-binding peptide, to NPs (CNP-Tβ4) [135]. Following its intravenous injection to mice post-ischemia and reperfusion, CNP-Tβ4 showed a greater fibrin targeting affinity and accumulation in the infarct myocardium than the control NPs, which led to greater efficacy in alleviating cardiac insufficiency and reducing scar size. This study suggests that targeting fibrin could be a potential therapeutic approach to delivering cardioprotective agents to damaged myocardium.

## 6. Cardiac-Specific Drug Delivery with NPs in Clinical Studies

Atherosclerotic plaque markedly increases the risk of thromboembolic events, particularly the occurrence of MI [136]. Shiozaki et al. developed a cholesterol-rich nanoemulsion (NE) to mimic the lipid composition and structure of low-density lipoprotein (LDL) [137]. Once LDL-NE makes contact with circulation, it attracts apolipoprotein E, which binds to the LDL receptors on the arterial endothelial cell membrane [138]. As a result, LDL-NE binds to the site of the atherosclerotic plaque, where there are abundant LDL receptors. The LDL-NE system was used to deliver paclitaxel to the atherosclerotic plaque site, thereby inhibiting macrophage migration, smooth muscle cell proliferation, and intimal invasion in the rabbit model of atherosclerosis [139]. In a clinical trial evaluating the impact of paclitaxel on patients with atherosclerosis, following the intravenous injection of paclitaxel-LDL-NE in patients with aortic atherosclerosis aged between 69 and 86 years, a reduction in the atherosclerotic lesion size was observed in half of the patients [NCT04148833] [137].

A new clinical trial aims to evaluate the safety and efficacy of an anti-inflammatory agent, methotrexate, in a cholesterol-rich non-protein NP (MTX-LDE) in patients with stable coronary disease [NCT04616872]. The LDE can bind to the LDL receptor of the atherosclerotic plaque by obtaining apolipoprotein. The patients were randomized to receive either the intravenous injection of MTX-LDE or placebo-LDE every 7 days for 12 weeks, and many plaque-related indicators, such as low attenuation plaque volume, noncalcified plaque volume, dense calcified plaque volume, total lumen value, total atheroma volume, and biochemical indexes were analyzed by coronary and aortic CT angiography. Although the trial has not been completed, the trial supports the concept that the modification of NPs with the lipid of LDL as a ligand for the atherosclerotic plaque is a promising system for cardiac-targeted drug delivery.

In contrast to intravenous injection, many clinical studies use intracoronary or intramyocardial administration as the delivery approach [140,141,142,143]. A NANOM FIM trial [NCT01270139] was performed with patients with stable angina, heart failure, atherosclerosis, and multivessel coronary artery disease. The patients’ circulating progenitor cells with silica-gold iron-bearing NPs were delivered to atherosclerotic lesions by endoscopic cardiac surgery. Twelve months after the delivery of the NPs, the total atheroma volume was significantly reduced in patients receiving NP-based delivery of progenitor cells [144].

## 7. Summary and Future Perspectives

We discussed the approaches for the NP-based cardiac-specific delivery of therapeutic agents for the treatment of ischemic heart diseases in preclinical and clinical studies. Although adeno-associated viral vectors (AAVs) have been shown to target cardiomyocytes, the low transfection efficiency, the time it takes for gene expression, and the lack of long-term gene expression are the major issues that limit its routine clinical use. On the contrary, as discussed above, the NP-based delivery of therapeutic agents holds the potential for tissue and cell type-specific targeting as well as the prolonged release of multiple agents for instant treatment. However, the application of NPs in the clinical treatment of cardiovascular diseases is relatively limited compared to other fields, such as tumors and neurological disorders. One of the major obstacles is a lack of specificity in the current cardiac-targeting systems. Future studies are warranted to identify specific ligands/receptors in the cardiomyocytes and develop novel NPs with high affinity and specificity.

## Figures and Tables

**Table 1:** Targeting Strategies for Nanomaterials Based Cardiac Specific Drug Delivery.

Targeted Location/Receptor	Nanocarrier	Therapeutic Agents	Animal Model	Outcomes	References
Cardiomyocytes/angiotensin II type 1 receptor (AT_1_R)	AT_1_-PEGylated-dendrigraft poly-L-lysine (DGL) DGL NPs	Anti-miR-1 antisense oligonucleotide (AMO-1)	C57BL/6 mice, MI model	Reduced cardiomyocytes apoptosis and infarct size	Xue et al. [65]
Cardiomyocytes/vascular cell adhesion molecule-1 (VCAM-1)	Polydopamine (PDA) core and a macrophage membrane shell NPs	Macrophage membrane shell	Sprague-Dawley rats, IR model	Reduced oxidation and pyroptosis in cardiomyocytes	Wei et al. [72]
Cardiomyocytes/CD63 antigen of exosomes or myosin-light-chain surface markers on cardiomyocytes	Fe_3_O_4_ core with a silica shell, and is decorated with poly (ethylene glycol) NPs	CD63-expressing exosomes	Rabbit and rat, MI model	Reduced infarct size and preserved left-ventricle ejection fraction and angiogenesis	Liu et al. [76]
Endocardium/atrial natriuretic peptide receptors	PEGylated porous silicon NPs	Atrial natriuretic peptide (ANP)	Wister rats, myocardial ischemia model	Attenuated hypertrophic signaling in the endocardium	Ferreira et al. [77]
Vascular Endothelium	Platelet membrane NPs	Poly (5,5-dimethyl-4,6-dithiopropylene glycol azelate) (PTK) and Cyclosporine (CsA)	Kunming mice, IR model	Reduced inflammation, reactive oxygen species, and myocardial fibrosis, improved left ventricular remodeling	Li et al. [81]
Vascular Endothelium	Platelet membrane vesicles	Carvedilol	Sprague-Dawley rats, IR model	Reduced cardiomyocyte apoptosis and infarct size, and preserved heart function	Zhou et al. [82]
Collagen-coated surfaces and endothelium-denuded aortas	Platelet nanovesicles	Cardiosphere-derived cardiac stem cells (CSCs)	Wistar-Kyoto (WKY) rats and adult farm pigs, IR model	Increased retention in the heart and reduce infarct size	Tang et al. [83]
Circulating monocytes	PLGA NPs	Irbesartan	C57BL/6 mice, IR model	Reduced infarct size and ameliorated ventricular remodeling	Nakano et al. [86]
Circulating monocytes	PLGA NPs	Pioglitazone	C57BL/6 mice and Chinese Bama mini pigs, IR model and MI model	Reduced inflammation and preserved cardiac healing	Tokutome et al. [87]

Note: MI: myocardial infarction; IR: ischemia and reperfusion.

## Data Availability

Not applicable.
